# Metabolic syndrome among overweight and obese adults in Palestinian refugee camps

**DOI:** 10.1186/s13098-018-0337-2

**Published:** 2018-04-19

**Authors:** Basma Damiri, Mohammed S. Abualsoud, Amjad M. Samara, Sakhaa K. Salameh

**Affiliations:** 1 Medicine & Health Science Faculty, Drug and Toxicology Division, An-Najah National University, Post Box 7, 0970 Nablus, West Bank Palestine; 2 Department of Medicine, An-Najah National University, Nablus, 0970 Palestine

**Keywords:** Metabolic syndrome, Obesity and overweight, Adults

## Abstract

**Background:**

Metabolic syndrome (MetS) is one of the main reasons for elevated cardiovascular morbidity and mortality worldwide. Obese and overweight individuals are at high risk of developing these chronic diseases. The aim of this study was to characterize and establish sex-adjusted prevalence of metabolic syndrome and its components.

**Methods:**

A cross-sectional study was conducted in 2015, 689 (329 men and 360 women) aged 18–65 years from three refugee camps in the West Bank. International Diabetes Federation and modified National Cholesterol Education Program-Third Adult Treatment Panel definitions were used to identify MetS.

**Results:**

The overall prevalence of obesity and overweight was high, 63.1%; Obesity (42 and 29.2% in women men; respectively and overweight 25.8 and 28.9% in women and men; respectively. The prevalence of MetS among obese and overweight was significantly higher (69.4%) according to IDF than NCEP definition (52%) (*p* < 0.002) with no significant differences between men and women using both definitions; (IDF; 71.8% men vs. 67.6% women, and (NCEP/ATP III; 51.9% men vs. 52.2% women). The prevalence of MetS increased significantly with increasing obesity and age when NCEP criterion is applied but not IDF. The prevalence of individual MetS components was: high waist circumference 81.3% according to IDF and 56.5% according to NCEP, elevated FBS 65.3% according to IDF and 56% according to NCEP, elevated blood pressure 48%, decreased HDL 65.8%, and elevated triglycerides 31.7%. Based on gender differences, waist circumferences were significantly higher in women according to both criteria and only elevated FBS was higher in women according to IDF criteria. Physical activity was inversely associated with MetS prevalence according to NCEP but not IDF. No significant associations were found with gender, smoking, TV watching, and family history of hypertension or diabetes mellitus.

**Conclusion:**

In this study, irrespective of the definition used, metabolic syndrome is highly prevalent in obese and overweight Palestinian adults with no gender-based differences. The contribution of the metabolic components to the metabolic syndrome is different in men and women. With the increase of age and obesity, the clustering of metabolic syndrome components increased remarkably. More attention through health care providers should, therefore, be given to the adult population at risk to reduce adulthood obesity and subsequent cardiovascular diseases.

**Electronic supplementary material:**

The online version of this article (10.1186/s13098-018-0337-2) contains supplementary material, which is available to authorized users.

## Background

The metabolic syndrome (MetS) refers to a compilation of several cardiovascular risk factors including obesity, hypertension, insulin resistance, and dyslipidemia [1]. The significance of diagnosing MetS is that it aids in identifying individuals at high risk of both cardiovascular diseases and type 2 diabetes [2]. In the presence of an epidemic of overweight and sedentary lifestyle; prevention, identification, and treatment of the MetS has become a major challenge for health care professionals [3].

Several expert groups attempted to produce diagnostic criteria for MetS in adults, and these sets of diagnostic criteria differ in terms of the cut-off points for each component [2, 4–6]. The National Cholesterol Education Program/Adult Treatment Panel III (NCEP/ATP III) recognized that the multiple components of the syndrome were cardiovascular risk factors and renamed the combination of these risk factors “The Metabolic Syndrome” [4]. The criteria The International Diabetic Federation (IDF) realized a defect in all the previous definition represented in the absence of ethnicity-specific cutoffs. This led to the production of a new definition for MetS in 2005 by the IDF, which addressed ethnic-specific values for waist measures, with straightforward, clinically useful diagnostic criteria [5].

Although experts are still facing the challenge of determining the cause of MetS, central obesity and insulin resistance are considered significant factors [7, 8]. This role led many researchers to conduct several studies to determine the prevalence of MetS among overweight and obese individuals [9]. Diabetes, overweight, and obesity had reached an alarming rate among Palestinians in the West Bank and Gaza [10–12]. The results vary depending on gender [13] or geographical and socio demographic factors [11]. According to the Palestinian Ministry of Health (MOH) annual reports in 2015, cardiovascular diseases were the leading cause of death and diabetes mellitus was the 4th cause of death among Palestinians [14]. The clinical significance of MetS is related to its impact on cardiovascular morbidity and mortality [15] which estimated to be about threefold more than those without the syndrome [15] and obese and overweight individuals in this population are at high risk for developing these chronic diseases [16].

A substantial body of literature had also demonstrated that living in socioeconomically disadvantaged areas is associated with an increased prevalence or incidence of MetS components [17]. Chronic psychological stress could be a major contributor to developing cardiovascular diseases, obesity, and ultimately the metabolic syndrome [18–23]. Palestinian refugees were displaced from the Palestinian area after the war 1948 to other Palestinian areas and have lived in camps in the West Bank (19 camps) and Gaza (8 camps) since 1949. They suffer from common daily life stressors such as high unemployment rate, low income, high poverty, poor infrastructure, and high population density [24, 25]. National studies had indicated that overweight and obesity had reached alarming rate among Palestinian refugees especially among women [10, 11] and therefore, they are at high risk of developing metabolic syndrome. As overweight, obesity, and their related diseases are largely preventable [26], a comprehensive understanding of MetS in adults’ population may be important for the specific direction of prevention strategies. Few studies had been conducted to establish the prevalence of MetS among overweight and obese adults in the West Bank and Gaza using IDF and NCEP criteria [27]. This research is a part of ongoing research that aim to establish the prevalence of MetS and to characterize its associated factors in overweight and obese Palestinians in different age groups living in different geographical areas and using both IDF and NCEP definitions. Therefore, this study specifically aimed to establish the prevalence of MetS in obese and overweight adults (18–65 year old) living in three refugee camps in Nablus, West Bank—Palestine using both the NCEP/ATP III and IDF definitions; and to characterize MetS components among them.

## Methods

### Study design and setting

A cross-sectional study was conducted in June 2015 in Nablus Districts in three refugee camps (Balata, Asker, and Al-Ein) in the north of Palestine. The study was conducted in United Nations Relief and Works Agency (UNRWA) clinics located in Nablus Governorate camps (Balata, Asker, and Al-Ein). Balata Camp is the largest refugee camp in northern West Bank (27,414 inhabitants) followed by Asker (19,408 inhabitants). Al-Ein camp is the smallest of the camps in Nablus and it has a population of 7903 inhabitants. Refugee camps were considered to participate if they have been residents of a specific camp for more than 6 months. Participants, who agreed to participate in the research and signed a consent form, were interviewed to answer the questionnaire questions [28] for personal, demographic, lifestyle, family history, and socioeconomic questions. The participant who presented with overweight and obesity (BMI ≥ 25) were invited to give blood samples. The following laboratory tests were carried out on peripheral blood: High-density lipoprotein (HDL), triglycerides, and fasting blood glucose. Candidates included in the study should be residents of Nablus city camps (Balata, Asker and Al-Ein) for at least the last 6 months, aged between 18 and 65 years, and do not meet the exclusion criteria. Subjects were excluded from the study if they had any of the following medical conditions: hypo- or hyper-thyroidism, Cushing syndrome, epilepsy, was taking regular medications other than anti-diabetic or anti-hyperlipidemia medications or refused to give a blood sample, who participated in the pilot study, and pregnant.

### Sampling

Based on the United Nations Relief and Works Agency (UNRWA), there were 54,727 residents in the three refugee camps in Nablus District (50% from Balata, 35% from Asker and 15% from Al-Ein). The percentage of adults was 59.2%, (50.9% males, and 49.1% females). There were 32,398 adults in the three camps. A stratified proportional sampling technique was used to select the subjects (people who met the inclusion criteria) from each camp (50% from Balata, 35% from Asker and 15% from Al-Ein). In the first stage, the population was divided to the three camps. The required sample size was 190 from Balata, 133 from Asker, and 57 from Al Ein. In the second stage, apparently, healthy adults who met the inclusion criteria were chosen from attendees of all UNRWA clinics in the three camps during 30 working days, 7 h/day. Every 3rd apparently healthy attendee was invited to participate in each camp. The response rate was 91%. A total of 689; 329 men and 360 women, Balata Camp (n = 372; 194 men and 178 women), Asker Camp (n = 203; 79 men and 124 women), Al Ein Camp (n = 114; 56 men and 58 women) had met the inclusion criteria and signed informed consent. They were interviewed to answer the questionnaire questions [28] for personal, demographic, lifestyle, family history, and socioeconomic questions. The participants who presented with overweight and obesity (435) were invited to give blood samples and 363 had accepted the invitation. The following laboratory tests were carried out on peripheral blood: HDL, triglycerides, and fasting blood glucose for 363 final participants.

### Diagnostic criteria

The NCEP/ATP III proposed that the individual must have at least three of the following cardiovascular risk factors: Fasting blood glucose > 110 mg/dL, blood pressure ≥ 130/85 mmHg, triglycerides ≥ 150 mg/dL, low HDL cholesterol (HDL-C) (men < 40 mg/dL, women < 50 mg/dL), waist circumference ≥ 102 cm in men or ≥ 88 cm in women [4]. To be diagnosed with MetS according to IDF criterion, the individual must have: Central obesity (defined as waist circumference ≥ 94 cm in men, ≥ 80 cm in women for Europid and respectively with ethnicity specific values for other groups) and at least two of the following: Raised fasting plasma glucose (FPG) ≥ 100 mg/dL or previously diagnosed type 2 diabetes, raised blood pressure: systolic BP ≥ 130 or diastolic BP ≥ 85 mm Hg, or treatment of previously diagnosed hypertension, raised triglycerides: ≥ 150 mg/dL or specific treatment for this lipid abnormality, reduced HDL cholesterol: < 40 mg/dL in men and < 50 mg/dL in women, or specific treatment for this lipid abnormality [5].

### Anthropometrics, blood pressure measurements, and biochemical analysis

Weight and height, for all participants (N = 689), were measured with the participant dressing light clothes and no shoes. The weight was reported to the nearest 0.1 kg and the height to the nearest 0.1 cm. Waist circumference was measured midway between the inferior margin of the thoracic cage and superior border of iliac crest during minimal inspiration. Any participant who had WC ≥ 102 for men and WC ≥ 88 for women according to NCEP/ATP III or WC ≥ 94 for men and WC ≥ 80 for women according to IDF was considered to have high WC. Body mass index (BMI) was calculated. After a good time of rest, blood pressure measurements were taken twice, 5 min apart, with the participant seated and the arm at the level of the heart, using standardized mercury sphygmomanometer (TXJ-10, China, and measures to the nearest 1 mmHg) with appropriate cuff size. The average of the two readings was used to record individual’s systolic and diastolic blood pressures. Participants who agreed to participate in the study and who were found to be obese or overweight (BMI ≥ 25) were asked if they were fasting—as the study was conducted in Ramadan- and insured that participants fasted for 8–12 h. Blood samples were collected and analysed for blood sugar, triglycerides, and HDL using “Roche Chemistry Analyser Cobas C 501, using alfa test kits (Alfa Wassermann B.V. Netherland)”.

### Measurement tools accuracy and precision assessment

Accuracy and precision of both anthropometric tools and the questionnaire were assessed; measuring tapes, the scale (EB9872, China, and measures to the nearest 0.1 kg). Interviews were conducted to avoid possible language and literacy issues. Researchers were trained for the interview process and a sample of 60 participants, 30 males and 30 women from the URWA clinics, was chosen for the pilot study.

### Data analysis

Statistical Product and Service Solutions (SPSS) (version 22, IBM Corporation) was used for data entry and analysis. Characteristics were described using means, standard deviations, and percentages wherever appropriate. The Pearson Chi square was used to compare the categorical variables. A p value of less than or equal 0.05 was considered statistically significant.

### Ethics

The study was carried out in accordance with the ethical standards, Declarations of Helsinki. Approval was obtained from Institutional Review Board “IRB” at An-Najah National University in Palestine prior to the research conduction. Approval of the director of UNRWA office in Nablus was taking prior to research conduction. All study participants were freely accepted to join the study and they provided a signed consent form. To insure privacy, interviews were carried out in private rooms. All participants were assured that all data collected will be confidential (using codes instead of names) and available for the research team only. It was explained to the participants that they had the right to withdraw from the research anytime. The questionnaire used in this study was obtained from Sirdah et al. research after obtaining permission from the author [28].

## Results

### General characteristics of study participants

In the first stage, 689; 329 men and 360 women, who met the inclusion criteria had participated, (50% from Balata Camp (n = 372; 194 men and 178 women), 35% from Asker Camp (n = 203; 79 men and 124 women), and 15% from Al Ein Camp (n = 114; 56 men and 58 men). The age distribution of the participants was as the following: 31% (n = 216) of study, participants were from age group (18–30), 22% (n = 159) from age group (31–40), 23% (n = 148) from age group (41–50), and 24% (n = 166) from age group (51–65) (Additional file 1).

### Sex-specific body mass index for all Participants (Stage 1)

The overall prevalence of obesity and overweight was (63.1; 58.1% in men and 67.8% in women). Obesity was more prevalent in women (42%) than men (29.2%) and overweight was more prevalent in men (28.9%) than women (25.8%). Both were statistically insignificant (Table 1). Out of 435 overweight or obese participants (191 men and 244 women), 363 (156 men and 207 women) accepted to participate in stage 2; (48.8% from Balata Camp (n = 177; 83 men and 94 women), 34.4% from Asker Camp (n = 125; 46 men and 79 women), and 16.8% from Al Ein Camp (n = 61; 27 men and 34 women). Out of 363 participants, 143 were overweight (74 men and 69 women), 129 with obesity type 1 (58 men and 71 women), 91 with obesity type 2 (24 men and 67 women).

**Table 1:** Sex-specific body mass index in all participants (Stage 1)

	Men no. (%)	Women no. (%)	Total no. (%)
Underweight (BMI ≤ 18.5)	12 (3.6)	7 (1.9)	19 (2.8)
Normal weight (BMI = 18.6–24.9)	126 (38.3)	109 (30.3)	235 (34.1)
Overweight (BMI = 25.0–29.9)	95 (28.9)	93 (25.8)	188 (27.3)
Obesity type 1 (BMI = 30.0–34.9)	70 (21.3)	79 (21.9)	148 (21.5)
Obesity type 2 (BMI ≥ 35.0)	26 (7.9)	72 (20.0)	98 (14.2)
Obese and overweight (BMI ≥ 25)	191 (58.1)	244 (67.8)	435 (63.1)
Obese and overweight agreed to participate in stage 2	156	207	363

### Prevalence of metabolic syndrome in obese and overweight based on NCEP/ATPIII vs. IDF definitions

Metabolic syndrome was significantly more prevalent when IDF definition was applied (69.4%) than NCEP**/**ATPIII (52.1%) (*p *= 0.002) (Table 2) with no significant differences between men and women using both definitions; (IDF; 71.8% men vs. 67.6% women, [*p* value 0.34 and *OR*: 1.218 (*CI* 0.773–1.919)] and NCEP/ATP III; 51.9% men vs. 52.2% women, (*p *= 0.96 and *OR* 0.99 (*CI* 0.653–1.501). With increasing obesity, the prevalence of MetS increased significantly according to NCEP criteria (*p *< 0.001) but not to the IDF criteria (*p *= *0.586*) (Table 3). According to IDF and NCEP criteria, the overall prevalence of metabolic syndrome was 62.3 and 74.8% in obese and 37.7 and 25.8% in overweight groups, respectively. According to NCEP definition, the prevalence of MetS had increased from 25.2% among overweight participants (BMI: 25–29.9) to 62% among obese (BMI: 30–34.9) and to 80% among obese type 2 (BMI > 35).

**Table 2:** Sex-specific prevalence of metabolic syndrome in obese and overweight based on NCEP/ATPIII vs. IDF definitions

Gender (n)	NCEP/ATPIII		IDF	
	With MetS no. (%)	Without Mets no. (%)	With MetS no. (%)	Without MetS no. (%)
Men (156)	81 (51.9)	75 (48.1)	112 (71.8)	44 (28.2)
Women (207)	108 (52.2)	99 (47.8)	140 (67.6)	67 (32.4)
Total (363)	189 (52.1)	174 (47.9)	252 (69.4)	111 (30.6)

**Table 3:** Prevalence of MetS based on different types of obesity according to both IDF and NCEP definitions

Type of obesity	IDF n (%)				NCEP n (%)			
	With MetS	Without	Total	p value	With MetS	Without MetS	Total	p value
Overweight	95 (37.7)	48 (43.3)	143 (39.4)	0.586	36 (25.2)	107 (74.8)	143 (39.4)	< 0.0001
Obesity type 1	93 (36.9)	36 (32.4)	129 (35.5)		80 (62.0)	49 (38.0)	129 (35.5)	
Obesity type 2	64 (25.4)	27 (24.3)	91 (25.1)		73 (80.2)	18 (19.8)	91 (25.1)	
Total	252 (69.4)	111 (30.6)	363 (100)	< 0.0001	189 (52.1)	174 (47.9)	363 (100)	0.431

### Prevalence of metabolic syndrome based on age -groups

Despite the definition used, the the prevalence of metabolic syndrome had increased by increasing age (Additional file 1) with significant increase between age groups when NCEP definition is applied (*p *< 0.0001). The prevalence ranged from 26.3% in age group 18–30 to 75.9 in the age group 51–65 according to NCEP criteria. When IDF criterion is applied, the prevalence had slightly increased with increasing age group (*p* = 0.37) and ranged from 61.4% in age group 18–30 to 69.4% in age group 51–65 years.

### Clustering of MetS components and sex-specific differences in metabolic abnormalities

**Fig. 1:**
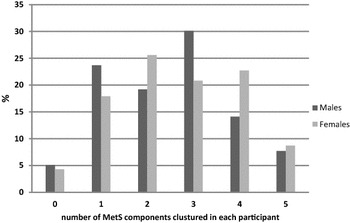
Clustering of the metabolic syndrome components in obese and overweight participants according to NCEP/ATP III criteria. The majority of (52.1%) have three or more components and only 4.7% lack all components of MetS. Sex-specific metabolic abnormalities in obese and overweight when the NCEP/ATP III criterion is applied had shown also variations in clustering MetS components

Most of the obese and overweight participants have increased central obesity (81.3%) according to IDF criterion (Table 4). The majority of women (n = 184, 88.9%) had increased central obesity compared to men (n = 111, 71.2%) (*p *< *0.0002*). Moreover, women with central obesity had shown increased (53.6%) clustering of metabolic abnormalities (2–3 components) compared to men (41.7%). Participants had shown variation in clustering of the MetS components based on gender when NCEP definition is applied (Fig. 1). The majority of the participants (52.1%) have three or more components and only 4.7% lack all components of MetS.

**Table 4:** Clustering of metabolic syndrome components in obese and overweight according to IDF definition

	Men no. (%)	Women no. (%)	Total no. (%)
Without MetS			
Normal WC	45 (28.8)	23 (11.1)	68 (18.7)
High WC only	6 (3.8)	8 (3.9)	14 (3.9)
High WC with 1 component	28 (17.9)	49 (23.7)	77 (21.2)
With MetS			
High WC with 2 components	33 (21.2)	54 (26.1)	87 (24.0)
High WC with 3 components	32 (20.5)	57 (27.5)	89 (24.5)
High WC with 4 components	12 (7.7)	16 (7.7)	28 (7.7)

*WC* waist circumference

Sex-specific prevalence of metabolic abnormalities in obese and overweight participants according to NCEP/ATP III and IDF citeria is shown in Table 5. Reduced HDL was the most prevalent abnormality in both men (64.1%) and women (67.1%). Moreover, high triglyceride was the least prevalent component in both men (35.9%) and women (28.5%). According to both criteria, women with central obesity were significantly more than men (*p *< 0.0002). Women with elevated blood glucose were significantly more than men according to IDF (*p* = 0.028) but not to the NCEP (*p *= 0.26). No statistical differences between men and women in all other abnormalities had been determined. Anthropometrics and biochemical characteristics of obese and overweight participants according to NCEP/ATPIII and IDF are shown in Additional file 2. There were significant differences in all anthropometrics and biochemical characteristics between participants with and without metabolic syndrome when NCEP/ATPIII criterion is applied (p < 0.001) in contrast to the IDF criterion that had only demonstrated significant difference in systolic blood pressure in women (*p *=* 0.037*).

**Table 5:** Sex-specific prevalence of metabolic abnormalities among obese and overweight participants according to NCEP/ATP III and IDF criteria

	All no. (%)	Men no. (%)	Women no. (%)	p value
WC (NCEP)	205 (56.5)	68 (43.6)	137 (66.2)	< 0.0002
WC (IDF)	295 (81.3)	111 (71.1)	184 (88.9)	< 0.0002
FBS (NCEP)	203 (56.0)	82 (52.6)	121 (58.8)	0.26
FBS (IDF)	237 (65.3)	92 (59)	145 (70)	0.028
BP	174 (48.0)	80 (51.3)	94 (45.4)	0.27
HDL	239 (65.8)	100 (64.1)	139 (67.1)	0.54
TG	115 (31.7)	56 (35.9)	59 (28.5)	0.13

*WC* waist circumference, *BP* blood pressure, *HDL* high density lipoprotein, *TG* triglycerides, *FBS* fasting blood sugar

### Socio-demographic factors and the prevalence of metabolic syndrome

Residency distribution for MetS using both IDF and NCEP is shown in Additional file 3. In general, there were no significant differences in overweight and obesity among refugees in the three camps but gender specific differences were observed. Overweight and obesity was significantly different in the three refugee camps among men but not women (*p* value 0.002) while increased WC was significantly different between the three camps among women (*p* value 0.02). The difference in the prevalence of MetS among the three camps was significant according the IDF (*p* value 0.000) but not according to NCEP (*p* value 0.29) criteria. In contrast to IDF that had demonstrated significant differences between those with metabolic syndrome and those without in geographic locality (*p *< 0.001) and marital status (*p *= 0.03), no differences had been demonstrated when the NCEP/ATPIII was applied (*p *> 0.05) (Table 6). The components of MetS varied also significantly depending geographical locality except for elevated triglyceride.

**Table 6:** Prevalence of metabolic syndrome according to socioeconomic and demographic factors using NCEP/ATPIII and IDF criteria

NCEP/ATPIII	With MetS (n = 189)	Without MetS (n = 174)	p value*
Residency			
Balata	88	89	0.297
Asker	72	53	
Al Ein	29	32	
Income			
Low	92	81	0.257
Intermediate	82	70	
High	15	23	
Marital status			
Married	167	148	0.353
Unmarried	22	26	

IDF	With MetS (n = 252)	Without MetS (n = 111)	p value*
Residency			
Balata	149	28	< 0.001
Asker	48	77	
Al Ein	55	6	
Income			
Low	129	44	0.075
Intermediate	101	51	
High	22	16	
Marital status			
Married	225	90	0.03
Unmarried	27	21	

* p value < 0.05 considered statistically significant. Chi square test was used

The prevalence of MetS according to different aspects of lifestyle using both criteria (NCEPT/ATPIII & IDF) is shown in Tables 7 and 8; respectively. No significant differences were found between participants with MetS and those without in relation to smoking, walking at least 1 h per day, practicing other additional sports, watching TV and/or setting on computer more than 4 h daily or family history of cardiovascular diseases, including hypertension (HTN), or diabetes (DM) (*p *> 0.05), when IDF criteria is applied. Similar results were found when applying the NCEP/ATP III criteria except for walking for more than 1 h daily OR: 0.53 (CI 0.35–0.81). The low cut off values for the IDF could explain the significant.

**Table 7:** Prevalence of MetS among obese and overweight based on modified NCEP/ATP III criteria, according to lifestyle

	With MetS (n = 189)	Without MetS (n = 174)	Odds ratio (CI)	p value*
Smoking				
Yes	55	60	0.78 (0.50–1.21)	0.271
No	134	114		
Walking ≥ 1 h/day				
Yes	76	97	0.53 (0.35–0.81)	0.003
No	113	77		
Additional sports				
Yes	15	19	0.70 (0.35–1.43)	0.330
No	174	155		
≥ 4 h/day of TV or computer				
Yes	63	61	0.93 (0.60–1.43)	0.729
No	126	113		
Family history of HTN/DM				
Yes	155	138	1.19 (0.71–2.0)	0.59
No	34	36		

* p value < 0.05 considered statistically significant. Chi square test was used

**Table 8:** Prevalence of MetS among obese and overweight based on IDF criteria, according to lifestyle

	With MetS (n = 252)	Without MetS (n = 111)	Odds ratio (CI)	p value*
Smoking				
Yes	84	31	1.29 (0.79–2.1)	0.308
No	168	80		
Walking ≥ 1 h/day				
Yes	123	50	1.16 (0.74–1.82)	0.508
No	129	61		
Additional sports				
Yes	25	9	1.25 (0.56–2.77)	0.585
No	227	102		
≥ 4 h/day of TV or computer				
Yes	86	38	1.00 (0.62–1.60)	0.984
No	166	73		
Family history of HTN/DM				
Yes	208	85	1.45 (0.84–2.50)	0.185
No	44	26		

* p value < 0.05 considered statistically significant. Chi square test was used

*HTN* hypertension, *DM* diabetes mellitus

## Discussion

The high prevalence of metabolic syndrome among people has challenged the medical community not to treat but to better identify people at risk. A comprehensive understanding of MetS in adults’ population may be important for the specific direction of prevention strategies. Cardiovascular diseases (CVD) are the leading cause of death and diabetes mellitus is the fourth cause of death among Palestinians in 2015 according to MOH [14]. The results of this study indicated that obese and overweight Palestinian refugee camps in the West Bank are at high risk for developing these diseases and this highlights the need for taking actions in order to treat these individuals and prevent the rest from developing the syndrome.

Obesity and insulin resistant are the principal causative factors in the development of MetS [7, 29]. The findings of this study had indicated that most of adult Palestinian refugees in Nablus have high central obesity according to IDF definition (81.3%) and NCEP definition (56.5%). Moreover, the overall overweight and obesity had reached alarming rate (63%) in this group (27.3% overweight and 35.7% obese) and significantly higher in women (67.8%) than men (58.1%). The majority of women (88.9%) had also increased central obesity compared to men (71.2%) and had shown significant increased (53.6%) clustering of metabolic abnormalities compared to men (41.7%). The prevalence of obesity, diabetes, and other cardiovascular risk factors was high, with central obesity and increased fast blood sugar being significantly higher in women and thus, putting them at greater risk for early mortality. These results agree with previous studies [11, 28, 30], and therefore, national health awareness and preventive programs should be established in order to decrease obesity and its associated diseases among refugees in Palestine especially among women.

The prevalence of MetS could vary according to age, gender, health status, and geographical location. Several studies had been conducted in the West Bank and Gaza in order to determine the prevalence of MetS and characterize its associated factors [13, 27, 28, 30–34]. Comparison between reported values is difficult as the prevalence varies widely according to different characteristics of populations and the criteria used by different investigators. The prevalence was established to be 17% among Palestinians in the West Bank [31] and 33.6% among Palestinians of East Jerusalem [30], 39.5% among Palestinians in Gaza Strip according to IDF and 23% according to NCEP [28, 33]. The prevalence was higher in non-healthy participants; 59.5% among clinic patients in Gaza Strip [34] using NCEP definition and 43.6% in patients with Schizophrenia in the West Bank [32]. A population prevalence was estimated to be 37% among Palestinian adults [13]. All these studies had demonstrated positive association between obesity and metabolic syndrome. To the authors’ best knowledge, there are few studies on MetS among people at higher risk; overweight and obese Palestinians [27]. Despite the definition used for metabolic syndrome in this study, the prevalence of MetS among overweight and obese adults in three refugee camps in Palestine was high; 52.1, and 69.4% using NCEP/ATP III and IDF criteria; respectively and had increased by increasing age and obesity. In comparison with the local studies, the high prevalence of obesity and overweight in this study may somewhat predict the high prevalence of metabolic syndrome in this population because obese individuals have a higher chance to develop the other components of MetS [35]. With increasing obesity, the prevalence of MetS increased using both criteria. According to NCEP definition, the prevalence of MetS had increased significantly from 25.2% among overweight participants to 62% among obese type 1–80% among obese type 2. In comparison with international studies, our results had reported higher frequencies of MetS among overweight and obese adult men and women than in the United States and seven European countries using NCEP/ATP III criteria. They found that the overall prevalence of MetS was 39% among obese and overweight groups [36]. Data from ten large cohort studies in seven European countries showed that the age-standardized percentage of obese participants with MetS ranged in women from 24 to 65%, and in men from 43 to 78% [9]. These high values should increase the attention of the health professionals, the media, and educational campaigns about the risks of obesity, especially central obesity, in developing coronary diseases and type 2 diabetes.

The most prevalent component of MetS among study participants was low HDL cholesterol (65.8%) followed by central obesity and elevated blood sugar (56.5, 56.0%; respectively) according to NCEP/ATP III criteria. It is known that low levels of HDL could be explained by the presence of secondary causes of low HDL, which are obesity [37], physical inactivity [38] and smoking [39] and associated with increased risk of coronary artery disease (CAD) [40]. Therefore, it is essential to increase the awareness in the population about methods of raising the concentration of HDL like weight loss, aerobic exercise, smoking cessation, and pharmacologic management with niacin and fibrates [41].

The differences in the prevalence of metabolic syndrome could be explained by genetic, lifestyle, and environmental factors. It was demonstrated that MetS strongly increases with age for both men and women [42, 43]. Similar to other studies, Our results had indicated that the prevalence of MetS increases with increasing age with 2.9 fold increase in the prevalence from age group 18–30 to age group 51–65 when the NCEP/ATP III criteria was implemented. This age related increase in the MetS prevalence highlights the vital role of raising awareness towards conducting screening at an early age. Similar trend was observed in different studies [3, 28, 44].

Several studies have shown strong associations between lifestyle and components of MetS, but most of these associations are still controversial [45–51]. Other lifestyle interventional studies have concluded that lifestyle modification is effective in improving MetS components [52]. As known, physical activity (PA) reduces risk factors related to metabolic syndrome and living in socioeconomically disadvantaged areas is associated with an increased prevalence or incidence of MetS components [53]. In this study, the prevalence of metabolic syndrome was significantly lower in participants who walk daily more than 1 h according to NCEP/ATP III criteria and they are 53% less likely to have MetS compared to participants with MetS. A concomitant finding was present in a study performed in Gaza Strip that also revealed a significant inverse relationship between walking and MetS prevalence [28]. The same inverse association between physical activity and MetS was found in other studies [47, 54–56]. This relationship could be explained by the role of physical activity in weight reduction which is a major component of MetS and thus could alter other components in an individual [47]. Adopting and promoting physical fitness programs by the Ministry of Health (MOH) could provide an effective primary prevention mean to reduce the prevalence of MetS, and thus reducing disastrous consequences of this syndrome such as strokes and heart attacks. Low levels of walking increase the likelihood of having MetS in both white and non-white older adults. The prevalence of metabolic syndrome was also compared with other lifestyle aspects including smoking, other type of sports, and sitting daily for more than 4 h on TV or computer or family history of cardiovascular diseases, including hypertension, or diabetes. Similar to other local studies [27, 28] no significant association was found between these aspects and the prevalence of metabolic syndrome when both NCEP/ATP III and IDF criteria were applied.

Chronic psychological stress, obesity, could be a major contributor to developing cardiovascular diseases and ultimately the metabolic syndrome [18–23]. The results of a previous study had indicated that the prevalence of MetS might be affected by different geographical areas in Palestine [13]. Another study had indicated that no worthy association was found between the geographical locality (city or refugee camp) and the prevalence of MetS among adult Palestinians in Gaza Strip [28]. Factors could explain the differences include different location, lifestyles, socioeconomic status, and chronic psychological stress. In general, most of the refugee camps in Palestine suffer from the same life stressors and have similar life style. Although we expected no differences between the three camps, our studies had demonstrated differences in the three camps in terms of MetS components and the prevalence of MetS according to IDF definition. Further investigation is recommended in order to determine these differences between the different locations.

To date, no uniform definition has been established to diagnose MetS among adults and more studies are recommended for each ethnicity. The diagnosing criteria for metabolic syndrome have been the subject of intense debate with such groups as the WHO, NCEP ATP III, IDF. In this study, the overall prevalence of MetS was significantly higher when IDF (69.4%) definition was applied than the NCEP (52.1%) with no significant differences between men and women using both criteria. Different studies had demonstrated that the IDF definition provided significantly higher prevalence of MetS among adult Palestinian than NCEP definition. The overall prevalence of MetS was also significantly higher using IDF definition than NCEP definition among adult Palestinians [33]. Moreover, ameta analysis was conducted for 8 Palestinian studies demonstrated that the IDF’s definition provided significantly higher prevalence (mean = 43.7%) of MetS in Palestinian adults compared to NCEP/ATP III’s definition (mean = 37.27%) and WHO’s definition (mean = 17%) [13]. The higher prevalence with the IDF compared to NCEP/ATP III can be explained by the higher cutoff values used in NCEP/ATP III, and the lower cutoff values of waist circumference as a prerequisite for the diagnosis using IDF criterion and for the elevated fast blood sugar [57, 58]. Although insufficient data about waist circumferences cutoff points have prevented region-specific definitions to be established for the Arabian population (IDF, 2006), IDF definition could be better than NCEP/ATP III for diagnosing MetS among Palestinian adults [33], however, follow up of people with MetS is indicated in order to identify which definition is a better predictor for CVD and DM.

## Conclusion

In this study and irrespective of the definition used, metabolic syndrome is highly prevalent in obese and overweight Palestinian adults with no gender-based differences. With the increase of age and obesity, the clustering of metabolic syndrome components was remarkably increased. This he high prevalence of MetS and its associated factors in adults live in refugee camps in Nablus city calls for an immediate intervention, given the fact that those adults are at risk of developing chronic diseases. Attention should be made through health care providers, social media, and educational campaigns about the benefits of losing weight, healthier diets, smoking cessation and increased physical activity in reducing MetS and its components [59].

## Additional files

**Additional file 1.** Prevalence of Metabolic Syndrome in Age- Adjusted Groups According to NCEP/ATP III and IDF.

**Additional file 2.** Anthropometrics and Biochemical Characteristics of Obese and Overweight Subjects According to NCEP/ATPIII and IDF.

**Additional file 3.** MetS Components in the Three Refugee Camps.

## Abbreviations

DM: diabetes mellitus
BMI: body mass index
BP: blood pressure
CI: confidential interval
cm: centimeter
CVD: cardiovascular diseases
FBS: fast blood sugar
FPG: fasting plasma glucose
HDL: high density lipoprotein
HTN: hypertension
IDF: International Diabetic Federation
IRB: Institutional Review Board
MetS: metabolic syndrome
Mg/dl: milligram per deciliter
MOH: Ministry of Health
mm Hg: millimeter of mercury
NCEP/ATP III: National Cholesterol Education Program/Adult Treatment Panel III
OR: odd ratio
SPSS: Statistical Product and Service Solutions
TG: triglycerides
TV: television
UNRWA: United Nations Relief and Works Agency
WC: waist circumferences
WHO: World Health Organization

## References

[1] Huang PL (2009). A comprehensive definition for metabolic syndrome. Dis Models Mech.

[2] Alberti KG, Zimmet PZ (1998). Definition, diagnosis and classification of diabetes mellitus and its complications. Part 1: diagnosis and classification of diabetes mellitus provisional report of a WHO consultation. Diabetic Med J Br Diabetic Assoc.

[3] Ford ES, Giles WH, Dietz WH (2002). Prevalence of the metabolic syndrome among US adults. JAMA.

[4] Third Report of the National Cholesterol Education Program (NCEP) expert panel on detection, evaluation, and treatment of high blood cholesterol in adults (Adult Treatment Panel III) final report. Circulation. 2002; 106(25):3143–421. 12485966

[5] Alberti KG, Zimmet P, Shaw J (2005). The metabolic syndrome—a new worldwide definition. Lancet (London, England).

[6] Balkau B, Charles MA (1999). Comment on the provisional report from the WHO consultation. European Group for the Study of Insulin Resistance (EGIR). Diabetic Med J Br Diabetic Assoc.

[7] Carr DB, Utzschneider KM, Hull RL, Kodama K, Retzlaff BM, Brunzell JD (2004). Intra-abdominal fat is a major determinant of the National Cholesterol Education Program Adult Treatment Panel III criteria for the metabolic syndrome. Diabetes.

[8] Furukawa S, Fujita T, Shimabukuro M, Iwaki M, Yamada Y, Nakajima Y (2004). Increased oxidative stress in obesity and its impact on metabolic syndrome. J Clin Investig.

[9] van Vliet-Ostaptchouk JV, Nuotio ML, Slagter SN, Doiron D, Fischer K, Foco L (2014). The prevalence of metabolic syndrome and metabolically healthy obesity in Europe: a collaborative analysis of ten large cohort studies. BMC Endocr Disord.

[10] Abdeen Z, Jildeh C, Dkeideek S, Qasrawi R, Ghannam I, Al Sabbah H (2012). Overweight and obesity among Palestinian adults: analyses of the anthropometric data from the first national health and nutrition survey (1999–2000). J Obes.

[11] El Kishawi RR, Soo KL, Abed YA, Muda WA (2014). Obesity and overweight: prevalence and associated socio demographic factors among mothers in three different areas in the Gaza Strip-Palestine: a cross-sectional study. BMC Obes.

[12] Shahin Y, Kapur A, Seita A (2015). Diabetes care in refugee camps: the experience of UNRWA. Diabetes Res Clin Pract.

[13] El Bilbeisi AH, Shab-Bidar S, Jackson D, Djafarian K (2017). The prevalence of metabolic syndrome and its related factors among adults in Palestine: a meta-analysis. Ethiop J Health Sci.

[14] Health Annual Report Palestine 2015. Palestinian Ministry of Health. 2015. http://www.moh.ps/Content/Books/NWNJXX7RJ92Bn4f5EGYiH43a2tjAAzKBnseGnEUCaqWqYZndsbCcPy_JQWguvkHTR4Xk4zUpdT45ooWxH11BhIbVAxwpGWy2wiwHdGcM5K7aZ.pdf. Accessed 14 Mar 2016.

[15] Isomaa PABO, Tuomi T, Forsén B, Lahti K, Nissén M, Taskinen MR, Groop L (2001). Cardiovascular Morbidity and Mortality Associated With the Metabolic Syndrome. Diabetes Care.

[16] Baumgartner RN, Heymsfield SB, Roche AF (1995). Human body composition and the epidemiology of chronic disease. Obesity.

[17] Krishnan S, Cozier YC, Rosenberg L, Palmer JR (2010). Socioeconomic status and incidence of type 2 diabetes: results from the black women’s health study. Am J Epidemiol.

[18] Carroll D, Phillips AC, Der G (2008). Body mass index, abdominal adiposity, obesity, and cardiovascular reactions to psychological stress in a large community sample. Psychosom Med.

[19] Chandola T, Brunner E, Marmot M (2006). Chronic stress at work and the metabolic syndrome: prospective study. BMJ.

[20] Dimsdale JE (2008). Psychological stress and cardiovascular disease. J Am Coll Cardiol.

[21] Matthews KA, Katholi CR, McCreath H, Whooley MA, Williams DR, Zhu S (2004). Blood pressure reactivity to psychological stress predicts hypertension in the CARDIA study. Circulation.

[22] Moore CJ, Cunningham SA (2012). Social position, psychological stress, and obesity: a systematic review. J Acad Nutr Dietetics.

[23] Vitaliano PP, Scanlan JM, Zhang J, Savage MV, Hirsch IB, Siegler IC (2002). A path model of chronic stress, the metabolic syndrome, and coronary heart disease. Psychosom Med.

[24] UNRWA. Balata Camp: united nations relief and works agency; 2018. https://www.unrwa.org/where-we-work/west-bank/balata-camp. Accessed 1 Mar 2018.

[25] UNRWA. Camp No. 1: united nations relief and works agency; 2018. https://www.unrwa.org/where-we-work/west-bank/camp-no-1-camp. Accessed 1 Mar 2018.

[26] Ofei F (2005). Obesity—a preventable disease. Ghana Med J.

[27] Damiri B, Aghbar A, Alkhdour S, Arafat Y (2017). Characterization and prevalence of metabolic syndrome among overweight and obese young Palestinian students at An-Najah National University. Diabetes Metab Syndr.

[28] Sirdah MM, Al Laham NA, Abu Ghali AS (2011). Prevalence of metabolic syndrome and associated socioeconomic and demographic factors among Palestinian adults (20–65 years) at the Gaza Strip. Diabetes Metab Syndr.

[29] Furukawa TFS, Shimabukuro M, Iwaki M, Yamada Y, Nakajima Y, Nakayama O, Makishima M, Matsuda M, Shimomura I (2004). Increased oxidative stress in obesity and its impact on metabolic syndrome. J Clin Invest.

[30] Abu Sham’a RA, Darwazah AK, Kufri FH, Yassin IH, Torok NI (2009). MetS and cardiovascular risk factors among Palestinians of East Jerusalem. East Mediterr Health J.

[31] Abdul-Rahim HF, Husseini A, Bjertness E, Giacaman R, Gordon NH, Jervell J (2001). The metabolic syndrome in the West Bank population: an urban-rural comparison. Diabetes Care.

[32] Sweileh WM, Zyoud SH, Dalal SA, Ibwini S, Sawalha AF, Ali I (2012). Prevalence of metabolic syndrome among patients with schizophrenia in Palestine. BMC Psychiatry.

[33] Sirdah MM, Abu Ghali AS, Al Laham NA (2012). The reliability of the National Cholesterol Education Program’s Adult Treatment Panel III (NCEP/ATP III) and the International Diabetes Federation (IDF) definitions in diagnosing metabolic syndrome (MetS) among Gaza Strip Palestinians. Diabetes Metab Syndr.

[34] Jamee YA, Abutawila H (2013). Risk factors of metabolic syndrome among clinic patients in Gaza-Palestine. Am J Cardiovasc Dis Res.

[35] Skinner AC, Perrin EM, Moss LA, Skelton JA (2015). Cardiometabolic risks and severity of obesity in children and young adults. N Engl J Med.

[36] Goodpaster BH, Krishnaswami S, Harris TB (2005). OBesity, regional body fat distribution, and the metabolic syndrome in older men and women. Arch Intern Med.

[37] Rashid S, Genest J (2007). Effect of obesity on high-density lipoprotein metabolism. Obesity (Silver Spring).

[38] Spate-Douglas T, Keyser RE (1999). Exercise intensity: its effect on the high-density lipoprotein profile. Arch Phys Med Rehabil.

[39] Dj H, Dm Y (2008). Targeting residual cardiovascular risk: raising high-density lipoprotein cholesterol levels. Heart.

[40] Victor H, Blaton IK, Bulo A (2008). How is metabolic syndrome related to dyslipidemia?. Biochem Med.

[41] Singh VN. Low HDL cholesterol (Hypoalphalipoproteinemia). http://emedicine.medscape.com/article/127943-overview. Accessed 19 June 2013.

[42] Hildrum B, Mykletun A, Hole T, Midthjell K, Dah AA (2007). Age-specific prevalence of the metabolic syndrome defined by the International Diabetes Federation and the National Cholesterol Education Program: the Norwegian HUNT 2 study. BMC Public Health.

[43] Mercedes R, Carnethon CML, Hill JO, Sidney S, Savage PJ, Liu K (2004). Risk factors for the metabolic syndrome. The coronary artery risk development in young adults (CARDIA) study, 1985–2001. Diabetes Care.

[44] Bjørn Hildrum AM, Hole T, Midthjell K, Dahl AA (2007). Ge-specific prevalence of the metabolic syndrome defined by the International Diabetes Federation and the National Cholesterol Education Program: the Norwegian HUNT 2 study. PMC Public Health.

[45] Lakka TA, Laaksonen DE, H-m Lakka, Männikkö N, Niskanen LK, Rauramaa R (2003). Sedentary lifestyle, poor cardiorespiratory fitness, and the metabolic syndrome. Med Sci Sports Exerc.

[46] Panagiotakos DB, Pitsavos C, Chrysohoou C, Skoumas J, Tousoulis D, Toutouza M (2004). Impact of lifestyle habits on the prevalence of the metabolic syndrome among Greek adults from the ATTICA study. Am Heart J.

[47] Park HS, Oh SW, Cho SI, Choi WH, Kim YS (2004). The metabolic syndrome and associated lifestyle factors among South Korean adults. Int J Epidemiol.

[48] Panagiotakos DB, Pitsavos C, Skoumas Y, Stefanadis C (2007). The association between food patterns and the metabolic syndrome using principal components analysis: the ATTICA Study. J Am Dietetic Assoc.

[49] Kastorini C-M, Milionis HJ, Esposito K, Giugliano D, Goudevenos JA, Panagiotakos DB (2011). The effect of Mediterranean diet on metabolic syndrome and its components: a meta-analysis of 50 studies and 534,906 individuals. J Am Coll Cardiol.

[50] Wada T, Urashima M, Fukumoto T (2007). Risk of metabolic syndrome persists twenty years after the cessation of smoking. Intern Med (Tokyo, Japan).

[51] Yokoyama H, Hiroshi H, Ohgo H, Hibi T, Saito I (2007). Effects of excessive ethanol consumption on the diagnosis of the metabolic syndrome using its clinical diagnostic criteria. Intern Med (Tokyo, Japan).

[52] Kalter-Leibovici O, Younis-Zeidan N, Atamna A, Lubin F, Alpert G, Chetrit A (2010). Lifestyle intervention in obese Arab women: a randomized controlled trial. Arch Intern Med.

[53] Vaughan C, Schoo A, Janus ED, Philpot B, Davis-Lameloise N, Lo SK, Laatikainen T, Vartiainen E, Dunbar JA (2009). The association of levels of physical activity with metabolic syndrome in rural Australian adults. BMC Public Health..

[54] Brien SE, Katzmarzyk PT (2006). Physical activity and the metabolic syndrome in Canada. Appl Physiol Nutr Metab Physiologie appliquee nutrition et metabolisme.

[55] Esteghamati A, Khalilzadeh O, Rashidi A, Meysamie A, Haghazali M, Abbasi M (2009). Association between physical activity and metabolic syndrome in Iranian adults: national surveillance of risk factors of noncommunicable diseases (SuRFNCD-2007). Metab Clin Exp.

[56] Strath S, Swartz A, Parker S, Miller N, Cieslik L (2007). Walking and metabolic syndrome in older adults. J Phys Act Health.

[57] Alberti KG, Zimmet P, Shaw J (2006). Metabolic syndrome—a new world-wide definition. A Consensus Statement from the International Diabetes Federation. Diabetic Med J Br Diabetic Assoc.

[58] Grundy SM, Cleeman JI, Daniels SR, Donato KA, Eckel RH, Franklin BA (2006). Diagnosis and management of the metabolic syndrome: an American Heart Association/National Heart, Lung, and Blood Institute Scientific Statement. Curr Opin Cardiol.

[59] NIH. How Is Metabolic Syndrome Treated?. http://www.nhlbi.nih.gov/health/health-topics/topics/ms/treatment. Accessed 3 Nov 2011.

