# Supplementary material for: Metabolic syndrome among overweight and obese adults in Palestinian refugee camps
Source: Diabetol Metab Syndr. 2018 Apr 19;10:34. doi: 10.1186/s13098-018-0337-2 (PMC5907715; doi:10.1186/s13098-018-0337-2)
Supplement: Supplementary file 1 — Additional file 1. Prevalence of Metabolic Syndrome in Age- Adjusted Groups According to NCEP/ATP III and IDF. [file 13098_2018_337_MOESM1_ESM.docx]

Additional File 1 Prevalence of Metabolic Syndrome in Age- Adjusted Groups According to NCEP/ATP III and IDF

| **NCEP /ATP III**  **Age groups in years** | | **With MetS**  **No. (%)** | **Without MetS**  **No. (%)** | **Total** |
| --- | --- | --- | --- | --- |
| **(18-30)** | Males | 8 (14) | 21 (36.8) | 29 (50.9) |
|  | Females | 7 (12.3) | 21 (36.8) | 28 (49.1) |
|  | **Total** | **15 (26.3)** | **42 (73.7)** | **57 (100)** |
| **(31-40)** | Males | 18 (21.7) | 24 (29.0) | 42 (50.6) |
|  | Females | 15 (18.1) | 26 (31.2) | 41 (49.4) |
|  | **Total** | **33 (39.8)** | **50 (60.2)** | **83 (100)** |
| **(41-50)** | Males | 18 (16.8) | 18 (16.8) | 36 (33.6) |
|  | Females | 35 (32.7) | 36 (33.6) | 71 (66.4) |
|  | **Total** | **53 (49.5)** | **54 (50.5)** | **107 (100)** |
| **(51-65)** | Males | 37 (31.9) | 12 (10.3) | 49 (42.2) |
|  | Females | 51 (44.0) | 16 (13.8) | 67 (57.8) |
|  | **Total** | **88 (75.9)** | **28 (24.1)** | **116 (100)** |
| **Total** | Males | 81 (22.3) | 75 (20.7) | 156 (43.0) |
|  | Females | 108 (29.8) | 99 (27.2) | 207 (57.0) |
|  | **All** | **189 (52.1)** | **174 (47.9)** | **363 (100)** |
| **IDF**  **Age groups** | |  |  |  |
| **(18-30)** | Males | 18 (31.6) | 11 (19.3) | 29 (50.9) |
|  | Females | 17 (29.8) | 11 (19.3) | 28 (49.1) |
|  | **Total** | **35 (61.4)** | **22 (38.6)** | **57 (100)** |
| **(31-40)** | Males | 30 (36.1) | 12 (14.5) | 42 (50.6) |
|  | Females | 26 (31.4) | 15 (18.0) | 41 (49.4) |
|  | **Total** | **56 (67.5)** | **27 (32.5)** | **83 (100)** |
| **(41-50)** | Males | 28 (26.2) | 8 (7.5) | 36 (33.7) |
|  | Females | 47 (43.9) | 24 (22.4) | 71 (66.3) |
|  | **Total** | **75 (70.1)** | **32 (29.9)** | **107 (100)** |
| **(51-65)** | Males | 36 (31.0) | 13 (11.2) | 49 (42.2) |
|  | Females | 50 (43.1) | 17 (14.7) | 67 (57.8) |
|  | **Total** | **86 (74.1)** | **30 (25.9)** | **116 (100)** |
| **Total** | Males | 112 (30.9) | 44 (12.1) | 156 (43.0) |
|  | Females | 140 (38.5) | 67 (18.5) | 207 (57.0) |
|  | **All** | **252 (69.4)** | **111 (30.6)** | **363 (100)** |
