# Supplementary material for: Metabolic syndrome among overweight and obese adults in Palestinian refugee camps
Source: Diabetol Metab Syndr. 2018 Apr 19;10:34. doi: 10.1186/s13098-018-0337-2 (PMC5907715; doi:10.1186/s13098-018-0337-2)
Supplement: Supplementary file 2 — Additional file 2. Anthropometrics and Biochemical Characteristics of Obese and Overweight Subjects According to NCEP/ATPIII and IDF. [file 13098_2018_337_MOESM2_ESM.docx]

Additional File 2 Anthropometrics and Biochemical Characteristics of Obese and Overweight Subjects According to NCEP/ATPIII and IDF

|  | With MetS  Mean(SD) | Without MetS  Mean (SD) | P-value* |
| --- | --- | --- | --- |
| **NCEP/ ATPIII Males** |  |  |  |
| Age | 47.1 (11.4) | 38.3 (11.8) | <0.001 |
| BMI | 32.9 (4.3) | 28.8 (3.3) | <0.001 |
| Waist circumference, cm | 106.9 (11.9) | 95.0 (9.0) | <0.001 |
| Systolic BP, mmHg | 132.5 (18.9) | 122.0 (18.1) | 0.001 |
| Diastolic BP, mmHg | 86.5 (10.2) | 79.9 (11.0) | <0.001 |
| HDL, mg/dl | 31.9 (9.9) | 42.4 (11.6) | <0.001 |
| TAG, mg/dl | 203.7 (134.8) | 102.1 (61.2) | <0.001 |
| Glucose, mg/dl | 140.8 (59.7) | 104.3 (26.4) | <0.001 |
| **NCEP/ ATPIII Females** | | | |
| Age | 49.6 (9.9) | 40.0 (10.5) | <0.001 |
| BMI | 34.9 (4.8) | 31.0 (5.0) | <0.001 |
| Waist circumference, cm | 100.5 (10.0) | 87.6 (11.0) | <0.001 |
| Systolic BP, mmHg | 133.2 (18.4) | 116.5 (16.4) | <0.001 |
| Diastolic BP, mmHg | 84.2 (9.5) | 75.2 (11.5) | <0.001 |
| HDL, mg/dl | 42.3 (11.9) | 48.3 (12.0) | <0.001 |
| TAG, mg/dl | 181.3 (138.2) | 83.3 (35.5) | <0.001 |
| Glucose, mg/dl | 163.4 (69.7) | 106.1 (33.5) | <0.001 |
| **IDF Males** |  |  |  |
| Age | 43.7 (12.1) | 40.7 (12.7) | 0.171 |
| BMI | 31.1 (4.4) | 30.5 (3.4) | 0.456 |
| Waist circumference, cm | 101.4 (12.3) | 100.5 (11.7) | 0.671 |
| Systolic BP, mmHg | 129.1 (19.2) | 123.2 (18.6) | 0.085 |
| Diastolic BP, mmHg | 83.9 (10.2) | 81.8 (13.0) | 0.300 |
| HDL, mg/dl | 36.1 (12.7) | 39.0 (9.5) | 0.167 |
| TAG, mg/dl | 164.8 (130.6) | 129.6 (68.2) | 0.093 |
| Glucose, mg/dl | 123.4 (53.5) | 122.8 (40.8) | 0.946 |
| **IDF Females** | | | |
| Age | 45.9 (10.9) | 43.1 (11.9) | 0.093 |
| BMI | 33.2 (5.4) | 32.7 (4.9) | 0.497 |
| Waist circumference, cm | 93.4 (12.1) | 96.2 (12.7) | 0.121 |
| Systolic BP, mmHg | 127.2 (20.9) | 121.2 (15.0) | 0.037 |
| Diastolic BP, mmHg | 81.0 (11.8) | 77.7 (10.2) | 0.058 |
| HDL, mg/dl | 44.7 (12.3) | 46.2 (12.2) | 0.423 |
| TAG, mg/dl | 125.1 (102.6) | 153.8 (132.8) | 0.090 |
| Glucose, mg/dl | 134.3 (63.2) | 139.6 (60.6) | 0.570 |

* P value < 0.05 considered statistically significant. T-test was used.
