# Supplementary material for: Metabolic syndrome among overweight and obese adults in Palestinian refugee camps
Source: Diabetol Metab Syndr. 2018 Apr 19;10:34. doi: 10.1186/s13098-018-0337-2 (PMC5907715; doi:10.1186/s13098-018-0337-2)
Supplement: Supplementary file 3 — Additional file 3. MetS Components in the Three Refugee Camps. [file 13098_2018_337_MOESM3_ESM.docx]

Additional File 3 MetS Components in the Three Refugee Camps

| Refugee Camp | BMI ≥25 | | | WC (NCEP) | | | WC (IDF) | | |
| --- | --- | --- | --- | --- | --- | --- | --- | --- | --- |
|  | Men | Women | Total | Men | Women | Total | Men | Women | Total |
|  | n(%) | n(%) | n(%) | n(%) | n(%) | n(%) | n(%) | n(%) | n(%) |
| Balata | 83(53.2) | 94 (45.5) | 177(48.8) | 36(43.3) | 53(56.4) | 89(50.3) | 58(69.9) | 78(83.0) | 136(76.8) |
| Askar | 46(29.5) | 79 (38.2) | 125(34.5) | 21(45.7) | 64(81.0) | 83(68.0) | 32(69.6) | 76(96.2) | 108(86.0) |
| Al Ein | 27(17.3) | 34 (16.4) | 61 (16.8) | 11(40.7) | 20(58.8) | 31(50.8) | 22(77.8) | 30(88.2) | 52(83.6) |
| P value | 0.004 | 0.572 | 0.402 | 0.981 | 0.002 | 0.067 | 0.705 | 0.02 | 0.97 |
| Total | 156 | 207 | 363 | 68 | 137 | 203 | 112 | 184 | 296 |

| Refugee Camp | FBS | Elevated BP | HDL | Elevated TG | MetS NCEP | MetS IDF |
| --- | --- | --- | --- | --- | --- | --- |
|  | n(%) | n(%) | n(%) | n(%) | n(%) | n(%) |
| Balata | 73 (44.1) | 99 (55.9) | 126 (71.2) | 46 (26.0) | 88(49.7) | 149(84.2) |
| Askar | 75 (65.6) | 52 (41.6) | 81 (64.3) | 47 (37.6) | 72(57.6) | 48(38.4) |
| Al Ein | 38 (70.5) | 23 (37.3) | 32 (52.2) | 22 (36.1) | 29(47.5) | 55(90.2) |
| P value | 0.000 | 0.01 | 0.02 | 0.074 | 0.29 | 0.000 |
| Total | 203 | 174 | 239 | 115 | 189 | 252 |
